# Revisiting the influence of learning in predator functional response, how it can lead to shapes different from type III

**DOI:** 10.1002/ece3.8593

**Published:** 2022-02-14

**Authors:** Octavio Augusto Bruzzone, María Belén Aguirre, Jorge Guillermo Hill, Eduardo Gabriel Virla, Guillermo Logarzo

**Affiliations:** ^1^ Instituto de Investigaciones Forestales y Agropecuarias Bariloche INTA and CONICET Bariloche Argentina; ^2^ Fundación para el Estudio de Especies Invasivas (FuEDEI) Hurlingham Argentina; ^3^ CONICET Ciudad Autónoma de Buenos Aires Buenos Aires Argentina; ^4^ Facultad de Agronomía y Zootecnia Universidad Nacional de Tucumán, San Miguel de Tucumán Tucumán Argentina; ^5^ Instituto de Entomología Fundación Miguel Lillo PROIMI‐Biotecnología CONICET San Miguel de Tucumán Tucumán Argentina; ^6^ Fundación para el Estudio de Especies Invasivas (FuEDEI) Hurlingham Argentina

**Keywords:** biological control, feeding interaction, functional response shape, learning curve, predator‐prey, prior experiences

## Abstract

Predator/parasitoid functional response is one of the main tools used to study predation behavior, and in assessing the potential of biological control candidates. It is generally accepted that predator learning in prey searching and manipulation can produce the appearance of a type III functional response. Holling proposed that in the presence of alternative prey, at some point the predator would shift the preferred prey, leading to the appearance of a sigmoid function that characterized that functional response. This is supported by the analogy between enzyme kinetics and functional response that Holling used as the basis for developing this theory. However, after several decades, sigmoidal functional responses appear in the absence of alternative prey in most of the biological taxa studied. Here, we propose modeling the effect of learning on the functional response by using the explicit incorporation of learning curves in the parameters of the Holling functional response, the attack rate (*a*), and the manipulation time (*h*). We then study how the variation in the parameters of the learning curves causes variations in the shape of the functional response curve. We found that the functional response product of learning can be either type I, II, or III, depending on what parameters act on the organism, and how much it can learn throughout the length of the study. Therefore, the presence of other types of curves should not be automatically associated with the absence of learning. These results are important from an ecological point of view because when type III functional response is associated with learning, it is generally accepted that it can operate as a stabilizing factor in population dynamics. Our results, to the contrary, suggest that depending on how it acts, it may even be destabilizing by generating the appearance of functional responses close to type I.

## Graphical abstract

In population dynamics, the association between learning and sigmoid functional response produces stability in predator‐prey dynamics. Prey switching is the most accepted cause but is incompatible with experimental data. Using a model with a power‐law learning curve, we found that learning also produces functional response curves which lead to instability.

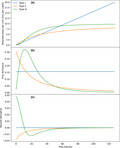

## 1 INTRODUCTION

Most biological systems involve an array of intricate relationships among organisms which are of paramount importance to understand the patterns of stability which according to May (1973), Gillman and Hails (1997), is the tendency of the system to stay or move away from the equilibrium, and biodiversity of communities (McCann, 2000). Therefore, it is crucial to count on reliable methods to have the best predictions and understanding of population and community dynamics, and eventually, to support wildlife management decisions (Pettorelli et al., 2015).

Functional response (Holling, 1959; Solomon, 1949) is a mathematical framework used to describe the ability of organisms to consume resources based on their availability. In this contribution, we will refer to carnivores, herbivores, parasites, parasitoids, hyperparasitoids, and some herbivores that consume the whole plant (such as phytophagous plankton that eat algae) as “predators.” The term “prey” here includes all different types of living organisms or food resources being consumed by the predator. As the survival of predators depends on their ability to exploit variable densities of prey, these organisms must be able to detect, process, and assimilate the prey as a function of its abundance; this ability is influenced by several factors. According to Holling (1966), the three basic components of the response of predators are (i) the attack rate (linked to the ability to find prey: *a*); (ii) the time prey is exposed: *t*; and (iii) the handling time (how fast a prey is consumed: *h*). In a classical paper, Holling (1959) characterized three types of functional response: type I response, in which the predator consumes its prey at a constant rate regardless of the prey density, and therefore it results in a linear relationship between prey density and consumption rate; the handling time is zero or near zero. Type II response (Holling’s disk equation), in which saturation occurs mostly because the handling time imposes a limit to the rate at which the prey is consumed, therefore it results in a rectangular hyperbola in which the rate of prey consumed asymptotically approaches 1/handling time. Finally, a type III response is a sigmoidal curve. The mathematical reason for the change of shape is that Holling’s disc equation now is a quadratic function of the prey density; the result is an “acceleration” of the attack rate, but it keeps the limitation caused by handling time. The component that produces this effect is learning (Holling, 1966). Real (1977) incorporated the possibility to shift between types II and III functional responses by using the enzyme kinetic models of Barcroft and Hill (1910). In Real’s approach, the attack rate depends on a Power‐Law of resource density as *a* = *bN^q^*, where *b* is the attack coefficient, *N* is the number of preys in the environment, and *q* is an exponent that influences the shape of the functional response from a hyperbolic type II functional response (*q* = 1) to a strict type III functional response (*q* = 2) and beyond these bounds.

The population consequences of each type of response are different, for instance, the stability of predator and prey populations strongly depends on whether predator consumption rates increase linearly (type I functional response) or follow a saturating function (type II and III functional responses) with prey densities (Hastings, 2013). Type III functional response is assumed to be able to stabilize predator‐prey systems (Hassell, 1978; Hassell et al., 1977; Murdoch & Oaten, 1975; Rall et al., 2008) since its lower efficiency at low prey densities would allow the prey population to recover from population bottlenecks and, in consequence, avoid local extinctions. While at high densities, it would increase the speed of consumption, helping to avert outbreaking‐type dynamics. However, the relationship between functional response types and stability is not simple. On the other hand, stability predictions differ depending on whether functional response parameters are derived. Several examples in predator‐prey systems were recorded for a type III functional response; however, the influence of learning on attack rate and handling time, and the consequence of these changes on the functional response, are poorly known.

According to Holling (1966), type III curves (S‐shaped) are indicative of organisms that show some form of learning behavior. These organisms have developed general responsiveness to many stimuli and can filter out irrelevant stimuli. Likewise, they can learn and separately channel information from different stimuli. These channels are not permanently established since the learned association will disappear unless it is reinforced or undergoes different experiences. The three key features of this behavior are *associative learning*, *information channeling*, and *forgetting* (Holling, 1966). Such features give organisms great flexibility which allows them to focus on a few stimuli and still retain the ability to take advantage of changes in the environment. In dynamic populations, when the prey density is very low, the predator might not associate this stimulus with a reward because the prey is so rare. Conversely, if prey density increases, the predator could become more responsive to the specific stimuli of the prey through learning. Tinbergen (1960) called this behavior the development of a specific searching image.

Holling’s model also reproduces prey switching, where the predator will consume preferentially (or more than proportionally), the most abundant prey. Thus, the predator will “switch” to another prey once the relative abundance of the different prey species reaches a critical threshold, which usually is near the inflection point in the sigmoidal functional response curve. Based on the enzyme kinetics equation, the shape of the curve is mediated by an *N* parameter which is the number of encounters that a predator must have with its prey before the predator is maximally efficient in consuming that prey. As the *N* term multiplies, the curve becomes increasingly more like a switch function (Real, 1979).

However, many predators do not have access to alternative prey due to their specificity, like some biological control agents (Byeon et al., 2011), or under laboratory conditions, they are exposed to only one type of prey; however, they exhibit a type III functional response quite frequently (Dunn & Hovel, 2020; Van Lenteren et al., 2016; Yazdani & Keller, 2016). Consequently, a different type of learning should take place, not mediated by the presence of alternative prey, but by the accumulated experience of the organism when searching, manipulating, and consuming prey. As in any learning process, the organism should then exhibit a learning curve (Shaw & Alley, 1985), in which the accumulated experience would translate into a modification of the functional response as a result of the experience.

Learning has been found extensively in almost all animal taxa (Manning & Dawkins, 1998; Shettleworth, 2001). These phenomena have long been described directly in parasitoid or predatory insects (Haverkamp & Smid, 2020; Little et al., 2019; Turlings et al., 1993; Vet et al., 1995). However, very few authors have studied how learning alters the parameters of the functional response curves, for example, Mendes et al. (2018) have found that, in egg predatory mites, the experienced females have significantly smaller manipulation times when compared to naive ones, but their attack rate is the same. Other authors investigated how pesticides affect predator efficiency, either because the predator attacks less prey or because of a decreasing searching time (He et al., 2012; Martinou & Stavrinides, 2015). These results show that learning can occur separately in attack rate or handling time, which makes the enzyme kinetic approach not fully compatible with the results of laboratory experiments or monophagous insects. Therefore, to explore the relationship between learning and functional response, an alternative model is necessary.

To test the hypothesis that the learning in predators produces the appearance of a type‐III functional response, in this study, we propose to explicitly incorporate learning curves in the parameters of the Holling’s disk equation (functional response type II) and to analyze what changes are produced in the functional response shape by applied learning on its fundamental parameters, the attack rate, and the handling time.

## 2 METHODS

### 2.1 Model

As a starting point, we used Holling’s disk equation of functional response type II:

(1) $$N=aDT/\left(1+ahD\right)$$

where *D* is the prey density, *a* the attack rate, *h* the handling time, *T* the length of the experiment, and *N* the consumed prey. The total consumed preys after a certain amount of time was called *N_t_*.

**FIGURE 1:**
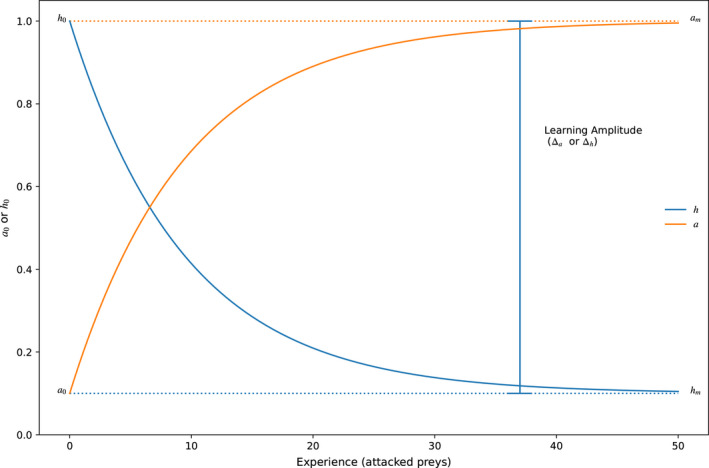
Theoretical learning curves proposed for the handling time (*h*) and the attack rate (*a*), both curves are of exponential learning type, where they tend exponentially to a final asymptotic value from an initial point. *h*_0_ and *a*_0_ are respectively the initial values of the handling times and the attack rate, while *h_m_* and *a_m_* are the final values of both variables. The learning amplitudes (*Δ_a_*, *Δ_h_*) are defined by the distances between the initial and final values of *h* or *a*

An exponential learning curve, based on Thurstone (1919) model and Estes (1950) statistical theory of learning in *a* and *h*, was added to the functional response type II model. Exponential is a very simple model in which the learning is proportional to the time (*T*) that take to do a given task *b*, in a differential equation form is *dT_b_*/*dN* = −*lT_b_*, as a consequence, according to Newell and Rosenbloom (1981), the learning occurs at a constant rate *l* as a function of the experience (here the number of consumed preys, *N*). By including this model, both parameters are allowed to improve as a function of preys attacked, resulting in a monotonic increase and decrease of *a* and *h*, respectively as shown in Figure 1.

For *a*, the model is a monotonically increasing function is:

(2) $$a\left(N\right)=a_{m}‐\left(a_{m}‐a_{0}\right)e^{‐laN}$$

$$\text{with}\;a>0\;\text{and}\;0\leq l_{a}\leq 1$$

where *l_a_* is the learning rate of *a* per attacked prey, and *a_m_* is the maximum possible attack rate for this species, with 0 < *a_m_* ≤ 1. Here the attack rate *a* increases asymptotically from the initial *a* at the beginning of the experiment (*a*_0_) to *a_m_* at a rate of *l_a_*. If *l_a_* = 0, there is no learning, but if *l_a_* = 1, the learning is maximum, and *a_m_* is approached after a single prey is consumed. How much it is possible to learn is *Δ_a_* = *a_m_*–*a*_0_.

A similar model was proposed for the handling time *h*, with the difference that in the case of *h*, it decreases with experience and asymptotically tends to zero instead of one as in the case of *a*, so

(3) $$h\left(N\right)=\left(h_{0}‐h_{m}\right)e^{‐l_{h}N}$$

$$\text{with}\;h_{m}\geq 0\;\text{and}\;h\geq 0$$

where *l_h_* is the learning rate of *h* per attacked prey, and *h_m_* is the minimum handling time for this species. The handling time *h* tends asymptotically from *h*_0_ (the handling time of the inexperienced predator) to *h_m_* at a rate of *l_h_*. How much it is possible to learn is *Δ_h_* = *h*_0_–*h_m_*. Both *Δ* (*Δ_a_*, *Δ_h_*) are called *learning amplitude*.

Finally, if *l_a_*, *l_h_*, *Δ_a_*, or *Δ_h_* are equal to zero there is no learning and the functional response function becomes Holling’s type II disk equation. The resulting dynamic of this model is shown in Figure 1, both *a* and *h* depend on the initial condition (*a*_0_, *h*_0_), the asymptotic values (*a_m_*, *h_m_*), the learning rate (*l_a_*, *l_h_*), and how much the predator can learn (*Δ_a_*, *Δ_h_*).

### 2.2 Prey depletion

The effect of prey depletion was also tested using the Rogers (1972) approach, in which the preys are a fixed pool in the experimental arena and are removed without reposition, so the differential equation is modified as:

(4) $$N=N_{0}\left(1‐e^{\left(‐Ta/\left(1+ahN\right)\right)}\right)$$

where *N*_0_ is the initial number of prey available, and *N* is the consumed prey, the available prey (*N_a_*) at time *T* is *N_a_* = *N*_0_–*N*. As a consequence, the available prey is constantly removed and its number decreases asymptotically toward zero.

### 2.3 Analysis

Under a learning context, the function would be expected to be convex at low prey densities, because the improvement in the predator’s ability to consume prey as a result of its experience is greater than its limitation in the ability to consume prey at a handling time greater than zero. At the inflection point, the improvement in the ability of the predator to consume prey as a result of learning is exactly compensated at that point by the limitation in the ability to consume prey, so the function becomes purely limited by the manipulation time.

**FIGURE 2:**
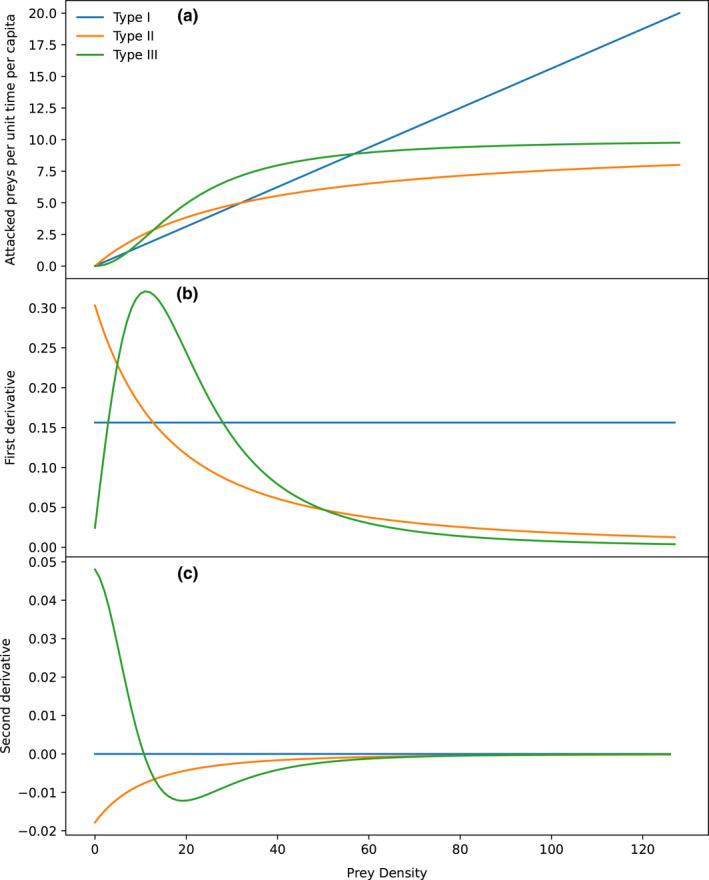
Theoretical functional response curves according to Holling’s (1959) paper, and its first and second derivatives, with the diagnostic characteristics of each type. Preys attacked as a function of preys offered for Holling’s three functional responses (a), (b) is the first derivative of the function, and below (c), the second derivative. The type III functional response can be characterized by the presence of an inflexion point in the slope of the curve, while the other two do not contain critical points of any kind

Therefore, a characteristic that allows identifying the type III functional response is its sigmoid convex‐to‐concave shape. There, the function of consumed preys (*N*) as a function of density (*D*) such as in any sigmoid function has an inflection point, so it is the second derivative zero. Before the inflection point, the function is convex (positive second derivative), and after this point, it is concave (negative second derivative). In type II functional response, the second derivative is always negative and asymptotically approaches zero, and on type I response, it is always zero, as it is a linear function (Figure 2). Therefore, the shape of the functional response was defined as the function of the second derivative of the functional response as a function of the offered preys. As explained above, the cases in which there was an inflection point in which the second derivative was zero while decreasing from positive to negative values were classified as type III, if it was always negative, they were classified as type II, and if it was zero or near‐zero, as type I. Additionally, if the slope of the second derivative was too low (near‐zero), and it was barely noticeable, they were considered *near*‐*type II*, and if the slope was low and always near zero, they were considered *near*‐*type I*. The three functional response functions with their corresponding first and second derivatives are shown in Figure 2.

To test whether learning in terms of improvement of attack rate and manipulation times generates type III functional responses, the analysis was aimed at identifying the different shapes of the functional response curve in different conditions of learning, here identified as parameters *l_a_* and *l_h_*. Other parameters affecting the behavior of the functional response curve were *a*_0_, *a_m_*, *h*_0_, and *h_m_*. To simplify the analysis, we assumed that *a_m_* = 1 and *h*_0_ = 1, so there are only four parameters to analyze, *l_a_*, *a*_0_, *l_h_*, and *h_m_*.

To avoid the *curse of dimensionality*, the parameters were tested in pairs, with the ones influencing the attack rate being analyzed separately from those influencing manipulation time. So one analysis was performed manipulating *a*_0_ and *l_a_*, keeping *h* constant (*l_h_* = 0, *h_m_* = *h*_0_) and another with *h_m_* and *l_h_* keeping *a* constant *(l_a_* = 0, *a_m_* = *a*_0_ *)*.

Time also influences the results, as the experiment became longer, the number of prey consumed and therefore the experience increased, so to keep the dimensions as low as possible, the length of the experiment was fixed to one time unit. As a consequence, T is removed from the equations as it is equal to one in a multiplication. Substituting the *a* and *l* parameters from the functional response Equation 1 by the Equations 2 and 3 will result in an equation where the *N* variable is present on both sides, as it happens with the Rogers random predator model from Equation 4, so we decided instead to solve numerically the models from Equations (1), (2), (3), (4) in the form of differential equations, as in Rosenbaum and Rall (2018) until the time equal one to find the results of this study. Equation 1, then became

(5) $$dN/dt=aDT/\left(1+ahD\right)$$

Equation 2 expressed as a differential equation is:

(6) $$da/dN=‐l_{a}\left(a_{m}‐a\right)$$

Equation 3 also expressed as a differential equation is:

(7) $$dh/dN=‐l_{h}\left(h‐h_{m}\right)$$

The Rogers random equation is simply the Holling’s disk equation with an exponentially decaying *N* substituted into it, so in its differential equation form it became:

(8) $$dN/dt=aN_{a}/\left(1+ahN_{a}\right)$$

and

$$dN_{a}=‐dN$$

where as in Equation 4, *N_a_* is the available prey, so *N* = *N*_0_ *– N_a_*.

The final form of the differential equations used for this study was for the parameters *a*_0_ and *l_a_*, the composition of Equations 1 and 2 into a system of ordinary differential equations:

(9) $$dN/dt=aD/\left(1+ahD\right)$$

$$da/dN=‐l_{a}\left(a_{m}‐a\right)$$

so, *a* is updated as a function of time using the number of prey consumed. In the prey‐limitation case, Equation 4 replaces Equation 1, and by replacing *dN* with −*dN_a_*, the sign of the equations is changed, as a result, the system changes to:

(10) $$dN_{a}/dt=‐aN_{a}/\left(1+ahN_{a}\right)$$

$$da/dN_{a}=l_{a}\left(a_{m}‐a\right)$$

$$\text{with}\;dN=‐dN_{a}$$

Similarly, the effect of *h_m_* and *l_h_* parameters were tested by composing Equations 1 and 3 into an ODE system:

(11) $$dN/dt=aD/\left(1+ahD\right)$$

$$dh/dN=‐l_{h}\left(h‐h_{m}\right)$$

And the effect of prey‐depletion became:

(12) $$dN_{a}/dt=‐aN_{a}/\left(1+ahN_{a}\right)$$

$$dh/dN_{a}=l_{h}\left(h‐h_{m}\right)$$

$$\text{with}\;dN=‐dN_{a}$$

### 2.4 Numerical methods

The tests were simulations at 1,002,001 combinations of parameters (a 1001 × 1001 matrix). For the attack rate *a*, the values used in the simulations were a range of *a*_0_ between 0.01 and 1.0 at intervals of 0.01, and with a range of *l_a_* between 0 (no learning) and 1 (maximum learning) at intervals also of 0.01. For the manipulation time *h*, the range of *h_m_* values was between 0.1 and 0 at intervals of 0.01, always starting from 1, and a range of learning rates *l_h_* as in *l_a_*, with a range between 0 and 1, also at intervals of 0.01. All the analyses were performed without and with prey limitation (Holling and Rogers models respectively). Each simulation was run to a fixed length of one time unit, and the prey densities were between 1 and 200 at intervals of 0.01. When the simulations were performed iterating over *l_a_* and *a*_0_, *l_h_* was considered zero (no learning in *h*), and *h* was fixed to 0.1; on the other hand, when the iterations were over *l_h_* and *h_m_*, *l_a_* was considered zero (no learning in *a*), and *a* was fixed to 0.1.

For each combination of parameters, the differential equations were solved by integrating numerically the differential equations using the fourth‐order Runge‐Kutta (RK4) integration method (Süli & Mayers, 2003), at steps of 1/1024 time units. All the simulations were performed with a series of routines written in the Python programming language which are available in Appendix S1.

## 3 RESULTS

### 3.1 Learning attack rate

**FIGURE 3:**
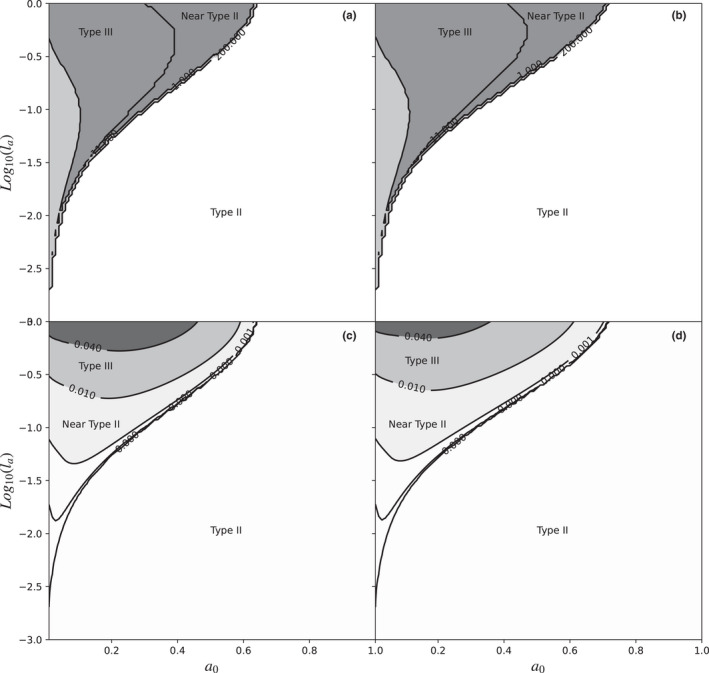
Functional response as a function of the combination between the logarithm of the learning rate (*l_a_*) on the Y‐axis, and the initial value of the attack rate (*a*_0_) on the X‐axis, without limitation by prey (a, c) and with limitation (b, d). As explained in the methods, the remaining parameters were *l_h_* = 0.0 (no learning), *h* = 0.1, length of the experiment = 1 time unit, and the range of prey densities used to calculate the derivatives were between 1 and 200 at steps of 0.01. In graphs a and b, the grayscales and contour lines show the prey density (*N*) at which the first inflexion occurs. Graphs c and d, on the other hand, show the maximum value of the second derivative of the functional response curve. Maximum values of second derivative greater than zero indicate type III functional responses, values close to zero, but positive, are near‐type II functional responses, finally, negative values indicate type II functional responses

The simulations showed that under learning that improves attack rates, the functional response was predominantly of type II, especially at low rates of learning (Figure 3), and at high levels of *a*_0_ (initial attack rate). On the other hand, at low levels of *a*_0_, the response began to transition from type II to type III responses, as the learning rate increased.

**FIGURE 4:**
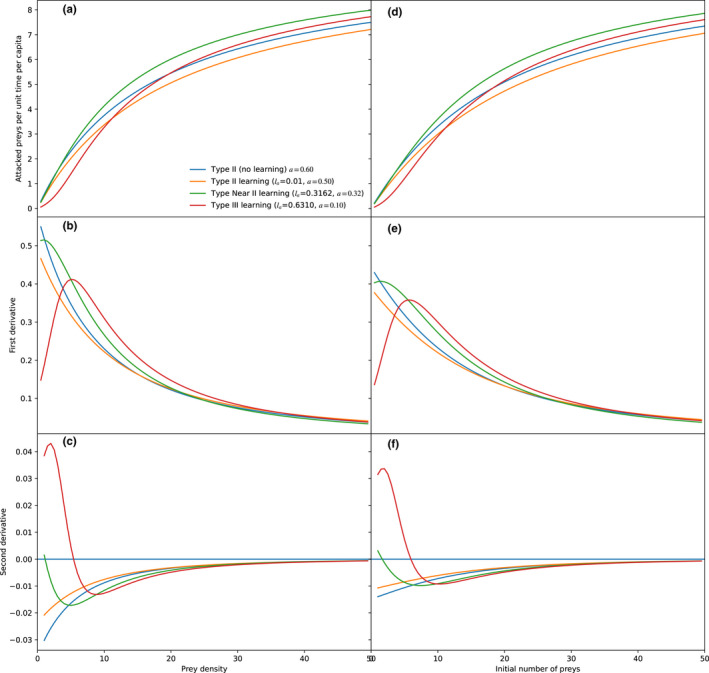
Consumed preys as a function of offered preys under different learning (*l_a_*) rates for attack rate (a, d), and first (b, e) and second derivatives (c, f). Plots a, b, and c are the models without prey depletion, so the X‐axis is the prey density, while d, e, and f are with depletion according to the Rogers model, therefore the X‐axis is the initial number of prey. With high learning rates, the functional response approaches type III functional response (positive second derivative at low prey density as a consequence of learning, and then negative as a consequence of handling time limitation). As explained in the methods, the remaining parameters were *l_h_* = 0.0 (no learning), *h* = 0.1, length of the experiment = 1 time unit

In an intermediate zone, the functional response was characterized as near‐type II, this response has characteristics of a type III response, such as a positive second derivative or close to zero, but very attenuated, so it is visually indistinguishable from type II (Figure 4).

At low initial attack rates (<0.3) and learning rates >0.1, the functional response is a Holling type III. Interestingly, when the learning rate becomes very high (>0.5), the near type II functional response again requires lower values of *a*_0_, since its inflection point becomes very close to zero in very low prey densities, so the curve becomes closer to type II at similar values of *a*_0_, but with lower learning values.

Under the Rogers model with prey depletion, the results are similar to those with constant density, the major difference is that the area in the parameter space in which the functional response is type III and/or close to II, is slightly larger, reflecting the effect of the reduction in the number of preys available in the shape of the functional response. On the other hand, the maximum second derivative under the Rogers model is lower than with the absence of prey limitation.

### 3.2 Learning handling time

**FIGURE 5:**
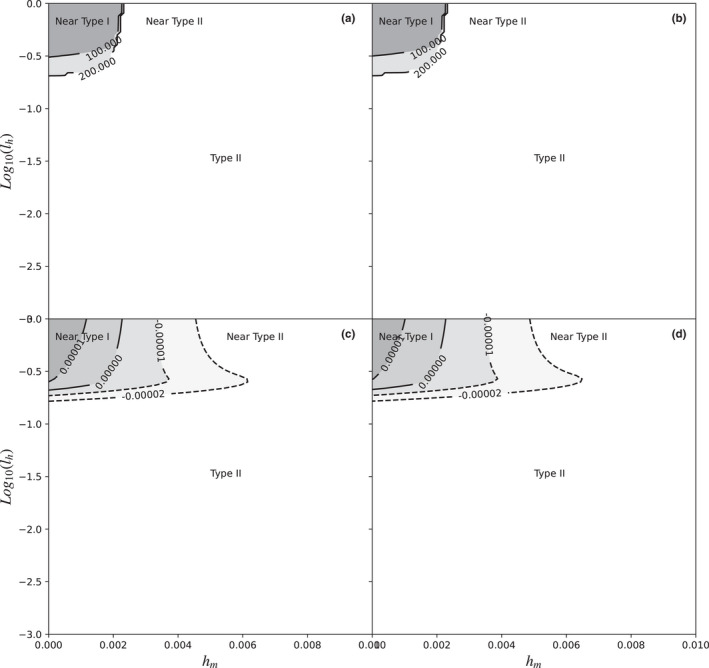
Functional response as a function of the combination between the logarithm of the learning rate (*l_h_*) on the Y‐axis, and the minimum handling time (*h_m_*) on the X‐axis without prey depletion (a, c) and with prey depletion (b, d). As explained in the methods, the remaining parameters were *l_a_* = 0.0 (no learning), *a* = 0.1, length of the experiment = 1 time unit, and the range of prey densities used to calculate the derivatives were between 1 and 200 at steps of 0.01. In graphs a and b, the grayscales and contour lines show the prey density (*N*) at which the first inflexion point occurs. Graphs c and d, on the other hand, show the maximum value of the second derivative of the functional response curve. Maximum second derivative values greater than zero indicate type I functional responses, values close to zero, but negative, are near‐type *II* functional responses, finally, negative values indicate type II functional responses

**FIGURE 6:**
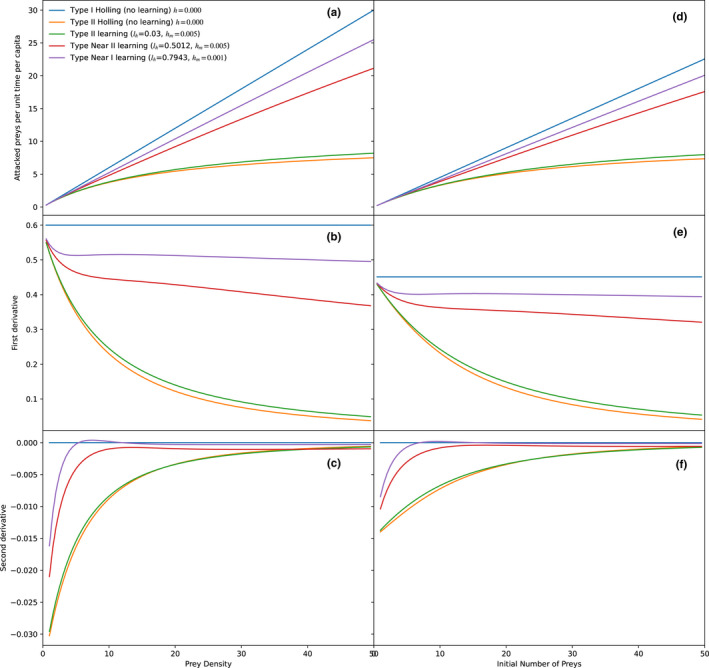
Consumed preys as a function of offered preys under different learning (*l_h_*) rates for handling time (a, d), and first (b, e) and second derivatives (c, f). Plots a, b, and c are the models without prey depletion, so the X‐axis is the prey density, while d, e, and f are with depletion according to the Rogers model, therefore the X‐axis is the initial number of preys. With high learning rates, the functional response approaches type I functional response (positive second derivative at low prey density as a consequence of learning, and then negative as a consequence of handling time limitation). As explained in the methods, the remaining parameters were *l_a_* = 0.0 (no learning), *a* = 0.1, length of the experiment = 1 time unit

Simulations carried out with manipulation time learning showed results where most of the combinations of parameters produce type II functional responses. Only in combinations of parameters with very high learning rates and very low minimum manipulation times the functional response begins to differ from type II to resemble type I (Figures 5 and 6), within the upper left corner of the said graph (*l_h_* = 1, and *h_m_* = 0), the functional response is type I.

As shown in Figure 6, the learning of the manipulation time never generated visually similar functional responses to type III; on the contrary, as the learning rate *l_h_* improved, the functional response looked more and more like type I. When the minimum handling time (*h_m_*) was very short, the curve equaled type I, otherwise, the curve resembled an intermediate between a truncated type I and type II. At low prey densities, the second derivative is always negative, only with very high learning rates (*l_h_* > 0.5), the second derivative becomes slightly positive at higher prey densities, to become slightly negative again. This does not conform to a type III functional response, but rather a near‐type I, because the derivative is very small, and the two critical points are unnoticeable. The effect of prey depletion was again small, with an area of type I functional response smaller than with Holling’s model.

## 4 DISCUSSION

Learning can produce all types of functional responses, depending on what parameters it affects. Under conditions of high learning amplitude, the functional response differed from type II. At low learning rates in both *a* and *h*, the result is a type II functional response, at high levels of learning in *a* prey‐predator is type III, while at high learning rates in *h*, the resulting functional response approaches type I, so if *h_m_* = 0, as the experiences accumulate, the handling time approaches asymptotically to zero, and the functional response approaches *dn*/*dt* = *aN*, which is the functional response type I. Learning can only produce type III functional responses if it affects the attack rate.

Theoretically, a linear functional response is possible when a predator can search and handle different prey simultaneously, or when the handling time is negligibly small (Hassell, 2000; Jeschke et al., 2002, 2004). A consequence of this work is that the type I functional response, which is usually interpreted as typical of filter feeders (Jeschke et al., 2004), or in general is not associated with learning processes, can be also a result of complex behaviors. Examples of type I functional responses have also been found in some parasitoid species (Kaçar et al., 2017; Mills & Lacan, 2004), and in filter‐feeding birds (Arzel et al., 2007), a taxon of animals capable of learning complex behaviors. Arzel et al. (2007) found a switch point between two types I functional responses with different slopes, showing that the complexity of the foraging behavior might imply several tasks that once optimized cannot be improved further and result in a curve with a series of discontinuities. Here we propose that this type of functional response is also a product of learning like type III, only that it is a different type since it has the same shape, but is caused by a different mechanism. So, it can be called type I_l_ if it is generated by learning, and type I_f_ it is generated by filter feeding. On the other hand, learning in terms of improvement in the attack rate would produce responses increasingly similar to III, while if the learning occurs in the optimization of handling times, a type I_l_ response would be generated.

Prey depletion did not change the overall pattern of the results. The main difference is that the maximum second derivative under the Rogers model is lower than with the absence of prey limitation, reflecting that as the available preys are reduced with time, so do the opportunities to learn, as a consequence the second derivative is smaller. Therefore, in an arena with a limited number of preys, the animals learn less, because they run out of preys. Here, the appearance of a turning point is earlier due to depletion of the prey and not due to learning. So, the functional response is more often type III, but not because of learning. The effect of handling time was similar with an area in which the functional response is type I or near‐type I, smaller than without prey depletion. The main reason is that as the number of available prey decreases with time, the functional response begins to be limited earlier, and keeps its functional response II shape even though its handling time decreases to a near‐zero value.

As proposed here, only the predator exhibits learning abilities, but prey may also learn, decreasing the effectiveness of predators (Brown, 2003; Zhao et al., 2006). Prey learning by personal experience is also costly because it involves surviving an encounter with the predator, so prey‐learning might resort also to social learning (Galef & Laland, 2005). Aggressive defensive behavior of preys might lead to complex patterns, including type‐IV functional response, in which the number of consumed preys decreases after reaching a maximum, product of prey collective defense actions (Líznarová & Pekár, 2013). To the best of our knowledge, no theoretical model is describing the effect of prey learning on the functional response, and the interactions between it and the predator abilities, so it remains an open topic in predator‐prey theory.

In terms of population dynamics, it is generally accepted that the type III functional response may have stabilizing effects on prey dynamics as proposed by Oaten and Murdoch (1975), the reason is the increase in the probability for a prey to be killed as their density increases, which means the presence of a positive second derivative on the functional response curve, as in the type III curve. Here we observed that this phenomenon (the transition from a type II response to a type III) occurs only in the case of strong learning in attack rate, but not in learning in handling time. However, the handling time learning produces a curve with an asymptote that increases with time and prey density, which can give a different type of stabilization that requires further more specific studies.

Another issue is that, since learning is an accumulated process, it will interact with population dynamics in the form of a delayed effectiveness response. For example, given that the prey population will decrease after a peak or outbreak as some herbivorous insects as described by Berryman et al. (1987). Under the approach used here, with cumulative learning, some predators might remain more effective for a while after a decrease in prey population. In the case of parasitoids, as the lifespan is the same as its hosts it is not an issue, while in the case of vertebrate insectivores it will cause further instability or local extinctions, by consuming more than proportionally when the population is small after an outbreak.

## 5 CONCLUSIONS

The results obtained in this study show that learning can change the functional response of predators in different ways since it is generally accepted in the literature by generating either type I, II, or III and intermediate forms in the absence of alternative prey. Therefore, learning can both be a stabilizing or destabilizing factor in the population dynamics, depending on which type of prey consuming behavior it affects.

## CONFLICT OF INTEREST

The authors declare no competing interests.

## AUTHOR CONTRIBUTIONS

**Octavio Augusto Bruzzone:** Conceptualization (lead); Formal analysis (lead); Investigation (lead); Methodology (lead); Software (lead); Supervision (lead); Writing – original draft (lead); Writing – review & editing (lead). **María Belén Aguirre:** Conceptualization (equal); Formal analysis (equal); Investigation (equal); Methodology (equal); Writing – original draft (equal); Writing – review & editing (equal). **Jorge Guillermo Hill:** Investigation (equal); Writing – original draft (equal); Writing – review & editing (equal). **Eduardo Gabriel Virla:** Conceptualization (equal); Supervision (lead); Writing – review & editing (lead). **Guillermo Logarzo:** Conceptualization (equal); Funding acquisition (lead); Resources (equal); Supervision (lead); Writing – review & editing (lead).

## Supporting information

Appendix S1

## Data Availability

The manuscript does not use any data.

## References

[ece38593-bib-0001] Arzel, C. , Guillemain, M. , Gurd, D. B. , Elmberg, J. , Fritz, H. , Arnaud, A. , Pin, C. , & Bosca, F. (2007). Experimental functional response and inter‐individual variation in foraging rate of teal (*Anas crecca*). Behavioural Processes, 75(1), 66–71. 10.1016/j.beproc.2007.01.001 17336000

[ece38593-bib-0002] Barcroft, J. , & Hill, A. V. (1910). The nature of oxyhaemoglobin, with a note on its molecular weight. The Journal of Physiology, 39(6), 411–428. 10.1113/jphysiol.1910.sp001350 16992995 PMC1533721

[ece38593-bib-0003] Berryman, A. A. , Stenseth, N. C. , & Isaev, A. S. (1987). Natural regulation of herbivorous forest insect populations. Oecologia, 71(2), 174–184. 10.1007/bf00377282 28312243

[ece38593-bib-0004] Brown, G. E. (2003). Learning about danger: chemical alarm cues and local risk assessment in prey fishes. Fish and Fisheries, 4(3), 227–234. 10.1080/10236249309378857

[ece38593-bib-0005] Byeon, Y. W. , Tuda, M. , Kim, J. H. , & Choi, M. Y. (2011). Functional responses of aphid parasitoids, *Aphidius colemani* (Hymenoptera: Braconidae) and *Aphelinus asychis* (Hymenoptera: Aphelinidae). Biocontrol Science and Technology, 21(1), 57–70. 10.1080/09583157.2010.521236

[ece38593-bib-0006] Dunn, R. P. , & Hovel, K. A. (2020). Predator type influences the frequency of functional responses to prey in marine habitats. Biology Letters, 16(1), 20190758. 10.1098/rsbl.2019.0758 31964265 PMC7013479

[ece38593-bib-0007] Estes, W. K. (1950). Toward a statistical theory of learning. Psychological Review, 57(2), 94–107. 10.1037/h0058559

[ece38593-bib-0008] Galef, B. G. , & Laland, K. N. (2005). Social learning in animals: empirical studies and theoretical models. BioScience, 55(6), 489–499.

[ece38593-bib-0009] Gillman, M. , & Hails, R. (1997). An introduction to ecological modelling: putting practice into theory (p. 202). Blackwell Science.

[ece38593-bib-0010] Hassell, M. P. (1978). The dynamics of arthropod predator‐prey systems. Monographs in Population Biology, 13, 1–237. http://europepmc.org/abstract/MED/732858 732858

[ece38593-bib-0011] Hassell, M. P. (2000). The spatial and temporal dynamics of host‐parasitoid interactions. Oxford University Press.

[ece38593-bib-0012] Hassell, M. P. , Lawton, J. H. , & Beddington, J. R. (1977). Sigmoid functional responses by invertebrate predators and parasitoids. The Journal of Animal Ecology, 46(1), 249–262. 10.2307/3959

[ece38593-bib-0013] Hastings, A. (2013). Population biology: Concepts and models. Springer Science & Business Media.

[ece38593-bib-0014] Haverkamp, A. , & Smid, H. M. (2020). A neuronal arms race: the role of learning in parasitoid‐host interactions. Current Opinion in Insect Science, 42, 47–54. 10.1016/j.cois.2020.09.003 32947014

[ece38593-bib-0015] He, Y. , Zhao, J. , Yu, Z. , Desneux, N. , & Wu, K. (2012). Lethal effect of imidacloprid on the coccinellid predator *Serangium japonicum* and sublethal effects on predator voracity and on functional response to the whitefly *Bemisia tabaci* . Ecotoxicology, 21, 1291–1300. 10.1007/s10646-012-0883-6 22447470

[ece38593-bib-0016] Holling, C. S. (1959). Some characteristics of simple types of predation and parasitism. The Canadian Entomologist, 91(7), 385–398. 10.4039/Ent91385-7

[ece38593-bib-0017] Holling, C. S. (1966). The functional response of invertebrate predators to prey density. Memories of the Entomological Society of Canada, 48, 1–86. 10.4039/entm9848fv

[ece38593-bib-0018] Jeschke, J. M. , Kopp, M. , & Tollrian, R. (2002). Predator functional responses: discriminating between handling and digesting prey. Ecological Monographs, 72(1), 95–112.

[ece38593-bib-0019] Jeschke, J. M. , Kopp, M. , & Tollrian, R. (2004). Consumer‐food systems: why type I functional responses are exclusive to filter feeders. Biological Reviews, 79(2), 337–349. 10.1017/S1464793103006286 15191227

[ece38593-bib-0020] Kaçar, G. , Wang, X.‐G. , Biondi, A. , & Daane, K. M. (2017). Linear functional response by two pupal *Drosophila* parasitoids foraging within single or multiple patch environments. PLoS One, 12(8), e0183525. 10.1371/journal.pone.0183525 28829796 PMC5567721

[ece38593-bib-0021] Little, C. M. , Chapman, T. W. , & Hillier, N. K. (2019). Considerations for insect learning in integrated pest management. Journal of Insect Science, 19(4), 6. 10.1093/jisesa/iez064 PMC6635889 31313814

[ece38593-bib-0022] Líznarová, E. , & Pekár, S. (2013). Dangerous prey is associated with a type 4 functional response in spiders. Animal Behaviour, 85(6), 1183–1190. 10.1016/j.anbehav.2013.03.004

[ece38593-bib-0023] Manning, A. , & Dawkins, M. S. (1998). An introduction to animal behaviour. Cambridge University Press.

[ece38593-bib-0024] Martinou, A. F. , & Stavrinides, M. C. (2015). Effects of sublethal concentrations of insecticides on the functional response of two mirid generalist predators. PLoS One, 10(12), e0144413. 10.1371/journal.pone.0144413 26641652 PMC4671552

[ece38593-bib-0025] May, R. M. (1973). Qualitative stability in model ecosystems. Ecology, 54(3), 638–641. 10.2307/1935352

[ece38593-bib-0026] McCann, K. S. (2000). The diversity–stability debate. Nature, 405(6783), 228–233. 10.1038/35012234 10821283

[ece38593-bib-0027] Mendes, J. A. , Lima, D. B. , Neto, E. P. D. S. , Gondim Jr, M. G. C. , & Melo, J. W. S. (2018). Functional response of *Amblyseius largoensis* to *Raoiella indica* eggs is mediated by previous feeding experience. Systematic and Applied Acarology, 23(10), 1907–1914.

[ece38593-bib-0028] Mills, N. J. , & Lacan, I. (2004). Ratio dependence in the functional response of insect parasitoids: evidence from *Trichogramma minutum* foraging for eggs in small host patches. Ecological Entomology, 29(2), 208–216. 10.1111/j.0307-6946.2004.00584.x

[ece38593-bib-0029] Murdoch, W. W. , & Oaten, A. (1975). Predation and population stability. In A. MacFadyen (Ed.), Advances in ecological research, Vol. 9 (pp. 1–131). Elsevier.

[ece38593-bib-0030] Newell, A. , & Rosenbloom, P. S. (1981). Mechanisms of skill acquisition and the law of practice. In J. R. Anderson (Ed.), Cognitive skills and their acquisition (pp. 1–55). Erlbaum.

[ece38593-bib-0031] Oaten, A. , & Murdoch, W. W. (1975). Functional response and stability in predator‐prey systems. The American Naturalist, 109(967), 289–298. 10.1086/282998

[ece38593-bib-0032] Pettorelli, N. , Hilborn, A. , Duncan, C. , & Durant, S. M. (2015). Chapter two ‐ individual variability: the missing component to our understanding of predator–prey interactions. In S. Pawar , G. Woodward , & A. I. Dell (Eds.), Advantages in Ecological Research ‐ Traitbased ecology from structure to function, Vol. 52 (pp. 19–44). Academic Press.

[ece38593-bib-0033] Rall, C. , Guill, C. , & Brose, U. (2008). Food‐web connectance and predator interference dampen the paradox of enrichment. Oikos, 117(2), 202–213. 10.1111/j.2007.0030-1299.15491.x

[ece38593-bib-0034] Real, L. A. (1977). The kinetics of functional response. The American Naturalist, 111(978), 289–300. 10.1086/283161

[ece38593-bib-0035] Real, L. A. (1979). Ecological determinants of functional response. Ecology, 60(3), 481–485.

[ece38593-bib-0036] Rogers, D. (1972). Random search and insect population models. The Journal of Animal Ecology, 369–383. 10.2307/3474

[ece38593-bib-0037] Rosenbaum, B. , & Rall, B. C. (2018). Fitting functional responses: Direct parameter estimation by simulating differential equations. Methods in Ecology and Evolution, 9(10), 2076–2090. 10.1111/2041-210X.13039

[ece38593-bib-0038] Shaw, R. E. , & Alley, T. R. (1985). How to draw learning curves: Their use and justification. In T. D. Johnston & A. T. Pietrewicz (Eds.), Issues in the ecological study of learning (1st edn., pp. 275–304). Lawrence Erlbaum. https://www.taylorfrancis.com/books/mono/10.4324/9781315802398/issues‐ecological‐study‐learning‐johnston‐pietrewicz

[ece38593-bib-0039] Shettleworth, S. J. (2001). Animal cognition and animal behaviour. Animal Behaviour, 61(2), 277–286. 10.1006/anbe.2000.1606

[ece38593-bib-0040] Solomon, M. E. (1949). The natural control of animal populations. Journal of Animal Ecology, 18(1), 1–35. 10.2307/1578

[ece38593-bib-0041] Süli, E. , & Mayers, D. F. (2003). An introduction to numerical analysis. Cambridge University Press.

[ece38593-bib-0042] Thurstone, L. L. (1919). The learning curve equation. Psychological Monographs, 26(3), i–51. 10.1037/h0093187

[ece38593-bib-0043] Tinbergen, L. (1960). The natural control of insects in pinewoods. I. Factors influencing the intensity of predation by songbirds. Archives Néerlandaises De Zoologie, 13(3), 265–343. 10.1163/036551660X00053

[ece38593-bib-0044] Turlings, T. C. L. , Wäckers, F. L. , Vet, L. E. M. , Lewis, W. J. , & Tumlinson, J. H. (1993). Learning of host‐finding cues by hymenopterous parasitoids. In D. R. Papaj & A. C. Lewis (Eds.), Insect learning (pp. 51–78). Springer. 10.1007/978-1-4615-2814-2_3

[ece38593-bib-0045] Van Lenteren, J. C. , Hemerik, L. , Lins, J. C. , & Bueno, V. H. P. (2016). Type II and III functional responses of three Neotropical mirid predators when exposed to a range of densities (4–256) of eggs of *Tuta absoluta* on tomato. Insects, 7(3), 34. 10.3390/insects7030034 PMC5039547 27420099

[ece38593-bib-0046] Vet, L. E. M. , Lewis, W. J. , & Carde, R. T. (1995). Parasitoid foraging and learning. In R. T. Cardé & W. J. Bell (Eds.), Chemical ecology of insects 2 (pp. 65–101). Springer.

[ece38593-bib-0047] Yazdani, M. , & Keller, M. (2016). The shape of the functional response curve of *Dolichogenidea tasmanica* (Hymenoptera: Braconidae) is affected by recent experience. Biological Control, 97, 63–69. 10.1016/j.biocontrol.2015.05.004

[ece38593-bib-0048] Zhao, X. , Ferrari, M. C. , & Chivers, D. P. (2006). Threat‐sensitive learning of predator odours by a prey fish. Behaviour, 1103–1121. 10.1163/156853906778607408

